# Association of dietary inflammatory index with sarcopenia in patients with Metabolic dysfunction-associated fatty liver disease: a cross-sectional study

**DOI:** 10.3389/fnut.2024.1486898

**Published:** 2024-11-08

**Authors:** Xianyao Wang, Rongjie Shi, Ying Zi, Jun Long

**Affiliations:** Department of Gastroenterology, The First Affiliated Hospital of Dali University, Dali, Yunnan, China

**Keywords:** dietary inflammatory index, Metabolic dysfunction-associated fatty liver disease, sarcopenia, NHANES, diet

## Abstract

**Background:**

Sarcopenia is a common complication of fatty liver, and sarcopenia increases the risk of advanced liver fibrosis in patients with Metabolic dysfunction-associated fatty liver disease (MAFLD). Chronic inflammation is the crucial link between sarcopenia and fatty liver. An anti-inflammatory diet is expected to be an essential measure to prevent sarcopenia in patients with fatty liver, and the dietary inflammatory index (DII) is a crucial tool for assessing the inflammatory potential of diets. However, the relationship between DII and sarcopenia in patients with fatty liver is unclear.

**Objective:**

This study investigated the correlation between the dietary inflammatory index (DII) and sarcopenia in patients with Metabolic dysfunction-associated fatty liver disease (MAFLD).

**Methods:**

Data for this study were obtained from the National Health and Nutrition Examination Survey (NHANES) 2017–2018, with 917 patients with MAFLD participating in the study. Participants were divided into three groups based on DII tertiles: group T1 (*n* = 305), group T2 (*n* = 306), and group T3 (*n* = 306), and binary logistic regression was used to assess the relationship between DII and sarcopenia with stratified analyses based on the weights recommended by the NHANES and multivariate linear regression was used to evaluate the association of DII with total appendicular lean mass.

**Results:**

After adjusting for all confounders, DII was significantly and positively associated with the risk of sarcopenia in women [OR: 1.61, 95% CI: (1.226, 2.06), *p* < 0.001]. The risk of sarcopenia was higher in the T3 group compared to the T1 group [OR: 4.04, 95% CI: (1.66, 9.84), *p* = 0.002]. DII was negatively associated with appendicular lean mass adjusted for body mass index in both men and women.

**Conclusion:**

DII was significantly associated with the risk of sarcopenia in female patients with MAFLD, with higher DII scores related to a higher risk of sarcopenia. Higher DII scores related to a higher risk of sarcopenia in men with significant fibrosis.

## 1 Introduction

Metabolism-associated fatty liver disease (MAFLD), a new diagnostic definition proposed in 2020 by an international panel of experts from 22 countries, emphasizes the metabolic dysregulation that accompanies fatty liver disease, previously known as non-alcoholic fatty liver disease (NAFLD), a leading cause of chronic liver disease worldwide, the prevalence of which has been increasing (1, 2). Sarcopenia is a progressive and generalized skeletal muscle disease involving accelerated loss of muscle mass and function (3). Globally, sarcopenia poses a huge challenge to human healthcare. Studies have shown that people with MAFLD are at higher risk of developing sarcopenia (4, 5). And sarcopenia increases the risk of advanced liver fibrosis and mortality in people with MAFLD (6–8). Therefore, prevention of sarcopenia in patients with MAFLD is essential.

It has been shown that MAFLD is associated with a systemic inflammatory response and that patients with MAFLD have elevated serum levels of interleukin-6 (IL-6), tumor necrosis factor-alpha (TNF-*α*), CC chemokine ligand 2 (CCL2), CC chemokine ligand 19 (CCL19) (9). Meanwhile, systemic chronic low-grade inflammation is involved in the development of sarcopenia (10). Given the link between inflammation and MAFLD and sarcopenia, anti-inflammatory interventions are expected to prevent sarcopenia in patients with MAFLD, and diet is one of the most important measures to control systemic inflammation. Diet is involved in inflammation, and dietary components such as total fat, trans fat, carbohydrate, and cholesterol can promote inflammation, and based on this, previous studies have developed the Dietary Inflammation Index (DII) for assessing the inflammatory potential of diets, with a high DII score being a marker of a pro-inflammatory diet and a lower DII score representing an anti-inflammatory diet (11). Studies have shown that the risk of sarcopenia increases as the DII increases (12). However, no studies have investigated the relationship between DII and sarcopenia in patients with MAFLD.

This study aimed to examine the correlation between DII levels and the risk of sarcopenia in patients with MAFLD, thereby providing a valuable reference for the prevention and management of sarcopenia in patients with MAFLD.

## 2 Methods

### 2.1 Study population

**Figure 1:**
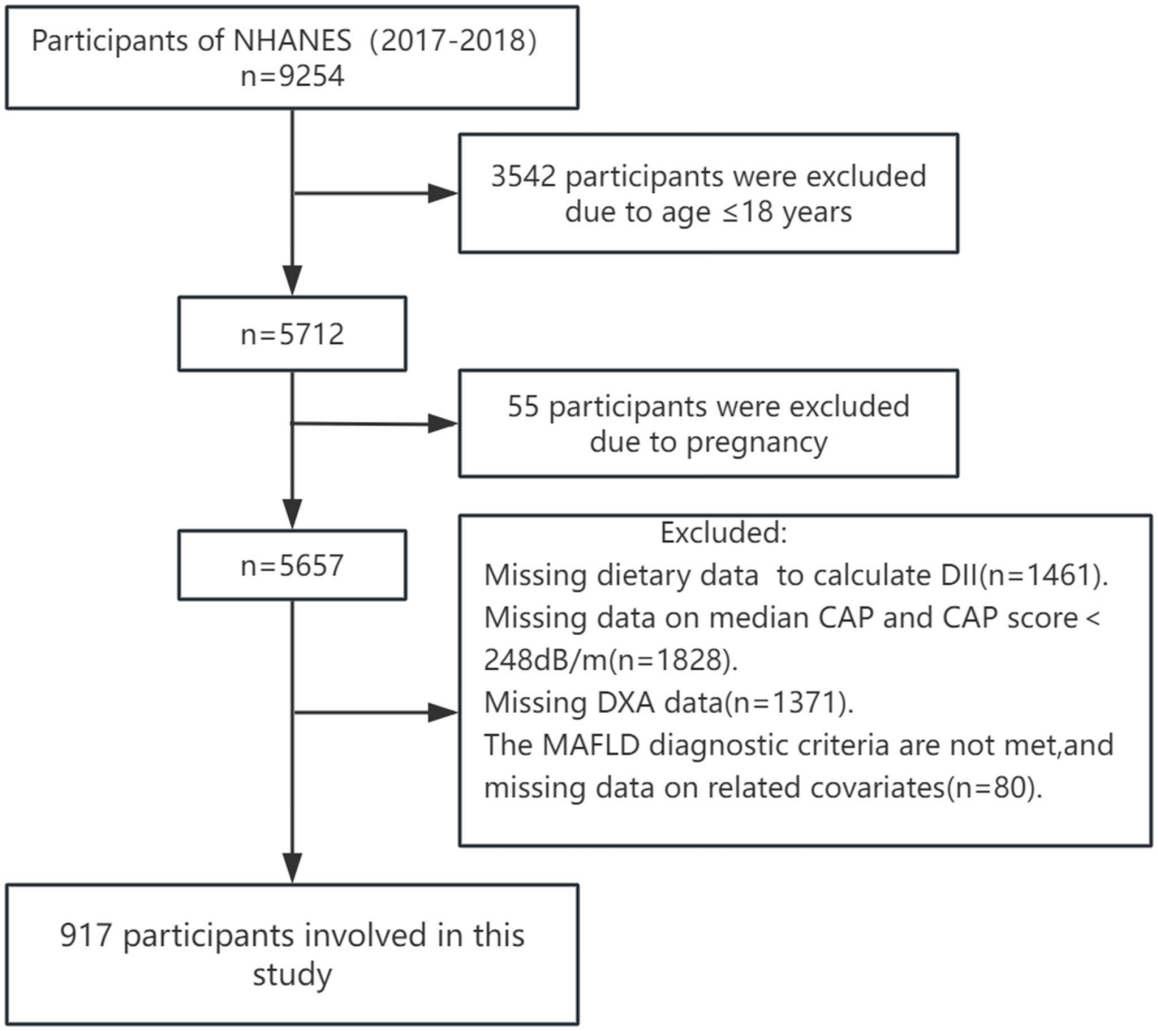
Flow chart of the study.

The National Health and Nutrition Examination Survey (NHANES) is the most in-depth survey administered by the National Center for Health Statistics (NCHS) to assess the health and nutritional status of adults and children in the United States. The NHANES surveys approximately 5,000 individuals annually in 15 different counties across the country in a two-year cycle, and the study cohort is representative of the entire U.S. population through a sample-weighted analysis. This study is based on data from the 2017 to 2018 NHANES, a cycle that included participants’ vibration-controlled transient elastography (VCTE) data used to define MAFLD. The Research Ethics Review Board of the National Center for Health Statistics approved the NHANES study. All participants provided informed consent. According to a large meta-analysis, a controlled attenuation parameter (CAP) of ≥248 dB/m (AUC: 0.823, Sensitivity: 0.688, Specificity: 0.822) was used as the threshold for the diagnosis of hepatic steatosis (13). And a median liver stiffness of ≥8.2 kPa was used to significant fibrosis (14). According to the European Association for the study of the Live (EASL) Clinical Practice Guidelines on non-invasive tests for evaluation of liver disease severity and prognosis, CAP≥275 dB/m might be used to diagnose steatosis (15). Therefore, we also conducted an analysis using the CAP ≥275 dB/m (Supplementary materials). Of the 9,254 participants, those who were not older than 18 years, pregnant, those with missing dietary data used to calculate DII, those with missing CAP data and CAP less than 248 dB/m, those with missing dual-energy X-ray data used to measure skeletal muscle mass, those who did not meet the diagnosis of MAFLD and those with missing data on relevant covariates were excluded, and finally, a total of 917 participants were enrolled in the study (Figure 1).

### 2.2 Definition of MAFLD

Based on the presence of imaging evidence of hepatic steatosis in combination with one of the following three conditions: overweight or obesity (defined as BMI ≥25 kg/m^2^ in Caucasians or BMI ≥23 kg/m^2^ in Asians), type 2 diabetes mellitus, and metabolic dysfunction. Metabolic dysfunction was defined as the presence of at least two of the following risk factors for metabolic abnormalities: (1) waist circumference ≥ 102 cm in Caucasian men and ≥ 88 cm in women (or ≥ 90 cm in men and ≥ 80 cm in women in Asians); (2) blood pressure ≥ 130/85 mmHg or specific drug treatment; (3) triglyceride (TG) ≥1.7 mmol/L or specific drug treatment; (4) high-density lipoprotein cholesterol (HDL-C) <1.0 mmol/L in men and HDL-C < 1.3 mmol/L in women or specific drug treatment; (5) prediabetes; (6) homeostasis model assessment of insulin resistance score (HOMA-IR) ≥2.5; and (7) high-sensitivity C-reactive protein (hs-CRP) >2 mg/L (1).

### 2.3 Definition of sarcopenia

According to the National Institutes of Health recommendations for determining the presence of sarcopenia, appendicular lean mass adjusted for body mass index (ALM_BMI_) is used. ALM_BMI_ = appendicular lean mass (kg)/body mass index (kg/m^2^), with males <0.789 and females <0.512 considered to have sarcopenia (16). The appendicular lean mass of the extremities was measured by dual-energy X-ray absorptiometry (DXA) whole-body scanning, which was obtained on a Hologic Discovery A optical densitometer (Hologic, Inc., Bedford, Massachusetts) using the Apex 3.2 software version. Trained and certified radiologic technologists perform DXA examinations and more detailed information on the DXA examination program is documented on the NHANES.^1^ In the NHANES files “DXDLLLE,” “DXDRLLE,” “DXDLALE” and “DXDRALE,” the specific values of limb lean body mass are recorded.

### 2.4 Calculation of DII

Daily intakes of dietary components in the NHANES database are obtained from 24-h dietary recall interview, and in this study, the average of two 24-h dietary data was used to calculate the DII. The specific calculation methodology is reported in detail elsewhere (11). In the present study, we used 28 different dietary components to estimate DII, including energy, protein, carbohydrate, dietary fiber, vitamins A, B1, B2, B6, B12, C, D, total fat, total saturated fatty acids, total monounsaturated fatty acids, total polyunsaturated fatty acids, n-3 fatty acids, n-6 fatty acids, cholesterol, vitamin E, *β*-carotene, niacin, folate, magnesium, iron, zinc, selenium, caffeine and alcohol.

### 2.5 Variables

Variables included in this study were gender, age, race, smoking, body mass index (BMI), Alcohol intake, Significant fibrosis, diabetes, cholesterol (TC), triglycerides (TG), high-density lipoprotein cholesterol (HDL-C), and glycosylated hemoglobin (HbA1c). Race was categorized as Mexican American, other Hispanic, Non-Hispanic White, Non-Hispanic Black, non-Hispanic Asian, and other races, BMI was weight (kg)/height (m) squared, and smoking was defined as smoking more than 100 cigarettes in one’s lifetime, which was obtained from a questionnaire. The methods of testing TC, TG, HDL-C, and HbA1c are described in detail on the official NHANES website. Diabetes mellitus was defined as “your doctor has told you that you have diabetes mellitus,” or a fasting blood glucose ≥7.0 mmol/L or a random blood glucose ≥11.1 mmol/L or an HbA1c >6.5%, or taking hypoglycemic medication to lower blood glucose or using insulin.

### 2.6 Statistical methods

Continuous variables were expressed as mean ± standard deviation and categorical variables were expressed as frequencies and weighted percentages, and weighted linear regression models (for continuous variables), as well as weighted chi-square tests (for categorical variables), were utilized to compare the differences between the two groups. Binary logistic regression was used to analyze the relationship between DII and sarcopenia. Model 1 was unadjusted for variables; model 2 was adjusted for age, race, and BMI; and model 3 was adjusted for age, race, BMI, Alcohol intake, smoking, significant fibrosis, diabetes, TC, TG, HDL-C and HbA1c. In addition, analyses were stratified by age, BMI, and significant fibrosis. Multivariate linear regression was used to estimate the relationship between DII and ALM_BMI_. Data were analyzed using the R package, EmpowerStats, and Stata, and *p* < 0.05 was considered statistically significant.

## 3 Results

### 3.1 Baseline characteristics of participants

In this study, 917 patients with MAFLD were enrolled with a weighted mean age of 42.58 years and a prevalence of sarcopenia of 13.07%. Participants were categorized into three groups based on DII tertiles: group T1 (*n* = 305), group T2 (*n* = 306), and group T3 (*n* = 306). There was a statistically significant difference in mean age between the three groups (T1: 44.56 ± 11.10 vs. T2: 41.86 ± 11.57 vs. T3: 41.24 ± 12.24, *p* < 0.001). In addition, participants with higher DII were more likely to be female (T1: 26.89% vs. T2: 54.12% vs. T3: 63.85%, *p* < 0.001), have a higher prevalence of diabetes mellitus (T1: 13.07% vs. T2: 12.29% vs. T3: 21.24%, *p* = 0.004), and a higher BMI (T1: 31.43 ± 5.67 vs. T2: 32.58 ± 6.55 vs. T3: 34.21 ± 6.94, *p* < 0.001), higher TC (T1: 4.84 ± 0.88 vs. T2: 5.08 ± 0.98 vs. T3: 5.21 ± 1.19, *p* = 0.009), lower alcohol intake (T1: 15.08 ± 24.75 vs. T2: 8.82 ± 24.59 vs. T3: 6.19 ± 14.46, *p* < 0.001), lower ALM_BMI_ (T1: 0.84 ± 0.17 vs. T2: 0.75 ± 0.18 vs. T3: 0.70 ± 0.17, *p* < 0.001). In these three groups, there were no statistical differences in smoking (*p* = 0.056), TG (*p* = 0.904), HDL-C (*p* = 0.941), HbA1c (*p* = 0.168), prevalence of significant fibrosis (*p* = 0.262) and prevalence of sarcopenia (*p* = 0.111). Detailed information is shown in Table 1. In female participants, we observed that DII was significantly higher in sarcopenia patients than in non-sarcopenia patients (*p* < 0.001), whereas there was no statistically significant difference in males (*p* = 0.568) (Table 2).

**Table 1:** The baseline characteristics of participants (weighted).

Variable	Total (*n* = 917)	T1 group (*n* = 305)	T2 group (*n* = 306)	T3 group (*n* = 306)	*p*-value
Age (years)	42.58 ± 11.72	44.56 ± 11.10	41.86 ± 11.57	41.24 ± 12.24	<0.001
Gender, n (%)					<0.001
Male	452 (52.09)	211 (73.11)	137 (45.88)	104 (36.15)	
Female	465 (47.91)	94 (26.89)	169 (54.12)	202 (63.85)	
Race/ethnicity, n (%)					0.003
Mexican American	185 (14.30)	76 (18.80)	63 (12.58)	46 (11.33)	
Other Hispanic	93 (8.69)	37 (10.56)	25 (6.88)	31 (8.65)	
Non-Hispanic White	268 (54.56)	70 (47.43)	92 (59.14)	106 (57.25)	
Non-Hispanic Black	162 (9.65)	38 (6.77)	55 (9.04)	69 (13.43)	
Non-Hispanic Asian	155 (6.74)	66 (8.30)	53 (6.65)	36 (5.14)	
Other Race	54 (6.07)	18 (8.15)	18 (5.71)	18 (4.20)	
Smoking, n (%)					0.056
Yes	345 (41.86)	106 (37.99)	113 (40.63)	126 (47.39)	
No	572 (58.14)	199 (62.01)	193 (59.37)	180 (52.61)	
Diabetes, n (%)					0.004
Yes	178 (15.39)	51 (13.07)	56 (12.29)	71 (21.24)	
No	739 (84.61)	254 (86.93)	250 (87.71)	235 (78.76)	
Significant fibrosis, n (%)					0.262
Yes	116 (12.26)	39 (11.99)	40 (10.31)	37 (14.65)	
No	801 (87.74)	266 (88.01)	266 (89.69)	269 (85.35)	
Alcohol intake (g)	10.12 ± 22.27	15.08 ± 24.75	8.82 ± 24.59	6.19 ± 14.46	<0.001
BMI, (Kg/m^2^)	32.71 ± 6.49	31.43 ± 5.67	32.58 ± 6.55	34.21 ± 6.94	<0.001
TC (mmol/L)	5.03 ± 1.02	4.84 ± 0.88	5.08 ± 0.98	5.21 ± 1.19	0.009
TG (mmol/L)	1.84 ± 1.87	1.79 ± 1.56	1.86 ± 1.71	1.88 ± 2.34	0.904
HDL-C (mmol/L)	1.27 ± 0.34	1.26 ± 0.32	1.28 ± 0.36	1.26 ± 0.34	0.941
HbA1c (%)	5.78 ± 1.10	5.68 ± 0.92	5.93 ± 1.28	5.76 ± 1.08	0.168
ALM_BMI_	0.77 ± 0.18	0.84 ± 0.17	0.75 ± 0.18	0.70 ± 0.17	<0.001
Sarcopenia, n (%)					0.111
No	770 (86.93)	266 (90.12)	255 (85.79)	249 (84.73)	
Yes	147 (13.07)	39 (9.88)	51 (14.21)	57 (15.27)	

Mean ± standard deviation was used to describe continuous variables, and unweighted frequencies and weighted percentages were used to describe categorical variables. *p*-values were calculated using weighted linear regression models for continuous variables and weighted chi-square tests for categorical variables.

**Table 2:** Comparison of DII by sarcopenia subgroups (weighted).

Variable	Women			Men		
	Non-sarcopenia	Sarcopenia	*p*-value	Non-sarcopenia	Sarcopenia	*p*-value
DII	1.42 ± 1.58	2.21 ± 1.51	<0.001	0.33 ± 1.84	0.46 ± 1.882	0.568

### 3.2 The association between DII and sarcopenia

The association between DII and the risk of prevalence of sarcopenia in NAFLD was analyzed using binary logistic regression models (Table 3). When DII was used as a continuous variable, it was significantly and positively associated with the risk of sarcopenia in women [model 1: odds ratio (OR): 1.42, 95% CI: (1.13, 1.78), *p* = 0.002]. This relationship remained statistically significant after adjusting for confounders [Model 2: OR: 1.57, 95% CI: (1.24, 1.99), *p* < 0.001. Model 3: OR: 1.62, 95% CI: (1.27, 2.08), *p* < 0.001]. In contrast, men had no significant correlation (*p* > 0.05). When DII was used as a categorical variable, in women, the T3 group had a higher risk of sarcopenia than the T1 group [Model 1: OR: 2.55, 95% CI: (1.09, 5.99), *p* = 0.031]. After adjusting for age, race, and BMI, the T3 group still exhibited a higher risk of sarcopenia [Model 2: OR: 3.95, 95% CI: (1.65, 9.46), *p* = 0.002]. After adjusting for age, race, BMI, Alcohol intake, smoking, significant fibrosis, diabetes, TC, TG, HDL-C and HbA1c, the association between DII and the risk of developing sarcopenia did not change [model 3: OR: 4.02, 95% CI: (1.64, 9.82), *p* = 0.002]. In men, a higher risk of prevalence of sarcopenia in the T2 group than in the T1 group was observed only in Model 2 and Model 3 [Model 2: OR: 2.48, 95% CI: (1.03, 5.96), *p* = 0.042. Model 3: OR: 2.87, 95% CI: (1.11, 7.41), *p* = 0.030].

**Table 3:** The association between DII and sarcopenia (weighted).

Variable	Model 1		Model 2		Model 3	
	OR (95%CI)	*p*-value	OR (95%CI)	*p*-value	OR (95%CI)	*p*-value
Women						
Continuous DII	1.42 (1.13, 1.78)	0.002	1.57 (1.24, 1.99)	<0.001	1.62 (1.27, 2.08)	<0.001
Categorical DII						
T1 group	1.00 (reference)		1.00 (reference)		1.00 (reference)	
T2 group	1.54 (0.62, 3.80)	0.348	1.84 (0.69, 4.92)	0.219	1.64 (0.59, 4.60)	0.344
T3 group	2.55 (1.09, 5.99)	0.031	3.95 (1.65, 9.46)	0.002	4.02 (1.64, 9.82)	0.002
Men						
Continuous DII	1.04 (0.86, 1.26)	0.670	1.04 (0.82, 1.33)	0.726	1.04 (0.82, 1.33)	0.724
Categorical DII						
T1 group	1.00 (reference)		1.00 (reference)		1.00 (reference)	
T2 group	1.97 (0.88, 4.44)	0.100	2.48 (1.03, 5.96)	0.042	2.87 (1.11, 7.41)	0.030
T3 group	1.58 (0.67, 3.73)	0.301	1.38 (0.42, 4.49)	0.596	1.41 (0.43, 4.60)	0.564

Model 1: Unadjusted variables.

Model 2: Adjusted for age, race, BMI.

Model 3: adjusted for age, race, BMI, Alcohol intake, smoking, significant fibrosis, diabetes, TC, TG, HDL-C and HbA1c.

### 3.3 Subgroup analysis

**Figure 2:**
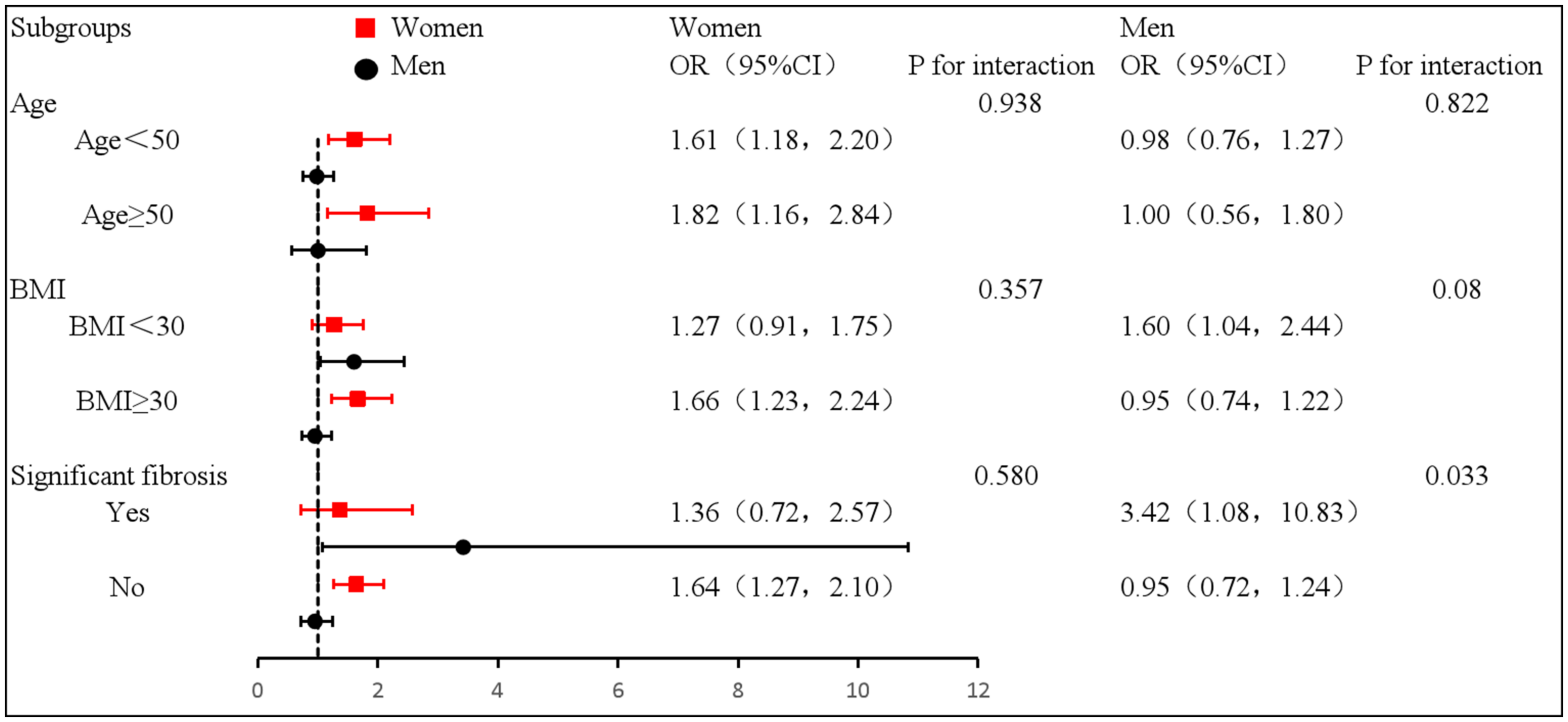
Forest plot of stratified analysis of the correlation between DII and the risk of sarcopenia in patients with MAFLD.

In a stratified analysis according to age (women: P for interaction = 0.938, men: P for interaction = 0.822), BMI (women: P for interaction = 0.357, men: P for interaction = 0.08) were stratified, and the risk of prevalence of DII and sarcopenia among MAFLD patients did not change. The association did not change and still showed a significant positive correlation between DII and the risk of sarcopenia in women, whereas in men, there was no significant correlation. However, after stratifying the participants according to significant fibrosis, higher DII scores related to a higher risk of sarcopenia in men with significant fibrosis [OR: 3.42, 95% CI: (1.08, 10.83), P for interaction = 0.033], and no significant difference among women (P for interaction = 0.580) (Figure 2).

### 3.4 The association between DII and ALM_BMI_

As shown in Table 4, multivariate linear regression analysis showed that DII was negatively associated with ALM_BMI_ in both men and women [women: Model 1: *β*: −0.008, 95% CI: (−0.014, −0.003), *p* = 0.004. Model 2: *β*: −0.007, 95% CI: (−0.012, −0.002), *p* = 0.010; men: Model 1: *β*: −0.007, 95% CI: (−0.013, −0.001), *p* = 0.042. Model 2: *β*: −0.007, 95% CI: (−0.013, −0.001), *p* = 0.049] (Table 4).

**Table 4:** Multivariate linear regression model between DII and ALMBMI (weighted).

ALM_BMI_	Model 1		Model 2	
	*β* (95%CI)	*p*-value	*β* (95%CI)	*p*-value
Women	−0.008 (−0.014, −0.003)	0.004	−0.007 (−0.012, −0.002)	0.010
Men	−0.007 (−0.013, −0.001)	0.042	−0.005 (−0.013, 0.001)	0.049

Model 1: Adjusted for age, race, BMI.

Model 2: adjusted for all remaining variables based on model 1.

## 4 Discussion

A total of 917 patients with MAFLD were included in this study, which showed that higher DII was significantly associated with the risk of developing sarcopenia in women, whereas no association was found in men. After stratification according to age and BMI, the association between DII and sarcopenia in patients with MAFLD was unchanged. However, after stratification according to significant fibrosis, higher DII scores related to a higher risk of sarcopenia in men with significant fibrosis, and no significant difference among women. These findings suggest that an anti-inflammatory diet may be an effective measure to prevent sarcopenia in patients with MAFLD.

Chronic low-grade inflammation throughout the body is a contributing factor to many chronic non-communicable diseases, and daily diet can influence the level of inflammation in the body. Poor dietary habits may promote the development of chronic inflammation, which in turn affects people’s health. The Mediterranean diet, which is rich in fruits, vegetables, whole grains, and olive oil, is considered an anti-inflammatory dietary pattern, and studies have shown that the Mediterranean diet reduces the level of inflammation in the body (17, 18). Meanwhile, the Mediterranean diet may positively affect biochemical parameters and fatty liver index in individuals with NAFLD (19). On the contrary, a diet high in fructose and fat may increase the level of inflammation in the organism (20). Experimental animal studies have shown that a high fructose diet for 8–12 weeks causes mice to develop fatty liver, with increased disease progression with longer exposure (21). A 6-week fructose-restricted diet (<7.5 g/meal and < 10 g/day) reduces intrahepatic lipid content (22). Therefore, a rational dietary profile can help to reduce the level of body inflammation. Currently, no one diet is the key for the treatment MAFLD, personalized approach maybe. DII can quantify diet-mediated inflammation and be used to assess the impact of dietary inflammation on disease. Studies have shown that higher DII is associated with the risk of developing several chronic diseases, including tumors (23–25), cardiovascular disease (26), Diabetes mellitus (27), Osteoporosis (28). The large prospective study by Petermann-Rocha et al. demonstrated that DII levels are associated with NAFLD severity (29). In addition, a cohort study found that high DII was significantly related to the incidence of NAFLD (30). However, the relationship between DII and sarcopenia in patients with fatty liver disease remains understudied. We associated DII with sarcopenia in patients with MAFLD and found that female patients with higher DII were more likely to develop sarcopenia. Previous studies have shown that higher DII is associated with an elevated risk of sarcopenia in patients with hypertension, asthma, chronic kidney disease, and Crohn’s disease, both in men and women (31–34). Our study showed this relationship only in women. This may be related to estrogen levels in female patients, which decrease as women age leading to muscle atrophy (35). However, in subgroup analyses stratified by significant fibrosis, we found that male patients with significant fibrosis are a particular population and that the higher the DII score, the higher the risk of sarcopenia in male patients with significant hepatic fibrosis. This finding has significant implications for the prevention and treatment of sarcopenia. We also analyzed the relationship between DII and appendicular lean mass. We found that DII was negatively associated with appendicular lean mass adjusted for body mass index. More high-quality studies in different subgroups still need to be added in the future to confirm the relationship between DII and sarcopenia in patients with MAFLD.

A chronic inflammatory state usually accompanies patients with MAFLD. In sarcopenia, the major pro-inflammatory cytokines include TNF-*α*, IL-6, and interleukin-1 (IL-1) (36). Chronic inflammation may be an essential factor in the development of sarcopenia in patients with MAFLD. Therefore, the mechanism by which a pro-inflammatory diet leads to the development of sarcopenia in patients with MAFLD may be related to inflammatory factors. Controlling the pro-inflammatory diet in patients with MAFLD may be an essential means of preventing sarcopenia. Increasing the intake of anti-inflammatory components (dietary fiber, vitamins, certain unsaturated fatty acids, etc.) and decreasing the intake of pro-inflammatory components (certain saturated fats, cholesterol, etc.) may be effective in preventing the development of sarcopenia in patients with MAFLD. However, the DII score is related to each nutrient component, and excessive control of the pro-inflammatory diet, which results in low intake of energy, protein, etc., may lead to malnutrition and thus loss of skeletal muscle, increasing the risk of sarcopenia (37). Adequate protein intake plays a vital role in ensuring muscle mass (38), and higher protein intake associated with lower prevalence of sarcopenia (39). Therefore, attention should be paid to energy and protein intake while controlling pro-inflammatory diets in patients with MAFLD. Data for this study were obtained from the National Health and Nutrition Examination Survey database, weighted according to officially recommended weights, and participants were representative of the entire U.S. population. Our study provides some valuable information on the dietary aspects of preventing sarcopenia in patients with MAFLD. It gives some reference for the prevention and control of MAFLD combined with sarcopenia. An anti-inflammatory diet may become one of the effective measures for the prevention of sarcopenia; therefore, we recommend that patients with MAFLD reduce the intake of pro-inflammatory dietary components and increase the intake of anti-inflammatory dietary components appropriately. However, the management of MAFLD combined with sarcopenia needs to place greater emphasis on a personalized approach and the acceptance of multiple possible diet solutions.

## 5 Limitations

The present study has some limitations; first, the dietary components used to calculate DII were obtained from a 24-h dietary recall interview, and recall bias is inevitable. Secondly, this study is a cross-sectional study, which can only conclude the correlation between DII and the occurrence of sarcopenia in the MAFLD population but cannot establish a causal relationship. In retrospective studies, dietary habits and environmental factors, etc., may not be able to match well with this population, so a large number of prospective studies are still needed in the future to confirm this conclusion.

## 6 Conclusion

The pro-inflammatory diet represented by higher DII scores was significantly associated with the risk of sarcopenia in female patients with MAFLD, with higher DII scores related to a higher risk of sarcopenia. Higher DII scores related to a higher risk of sarcopenia in men with significant fibrosis. DII was negatively correlated with body mass index-adjusted skeletal muscle mass in the extremities. A high DII score is a risk factor for sarcopenia in female patients with MAFLD.

## Data Availability

The datasets presented in this study can be found in online repositories. The names of the repository/repositories and accession number(s) can be found at: https://www.cdc.gov/nchs/nhanes/index.htm.

## Footnotes

^1^ https://www.cdc.gov/nchs/nhanes/index.htm
