# Supplementary material for: Association of dietary inflammatory index with sarcopenia in patients with Metabolic dysfunction-associated fatty liver disease: a cross-sectional study
Source: Front Nutr. 2024 Nov 8;11:1486898. doi: 10.3389/fnut.2024.1486898 (PMC11583804; doi:10.3389/fnut.2024.1486898)
Supplement: Supplementary file 1 [file Table_1.DOCX]

**CAP≥275 dB/m**

**we have added the analysis of this cut-off value.**

The baseline characteristics of participants (weighted).

| Variable | Total（n=690） | T1 group（n=305） | T2 group（n=306） | T3 group（n=306） | P-value |
| --- | --- | --- | --- | --- | --- |
| Age（years） | 42.56±11.66 | 44.01±11.20 | 42.12±11.47 | 41.55±12.18 | 0.065 |
| Gender，n（%） |  |  |  |  | ＜0.001 |
| Male | 361（55.03） | 158（75.12） | 117（49.73） | 86（40.21） |  |
| Female | 329（44.97） | 64（24.88） | 119（50.27） | 146（59.79） |  |
| Race/ethnicity，n（%） |  |  |  |  | 0.034 |
| Mexican American | 154（15.86） | 60（20.55） | 54（14.18） | 40（12.89） |  |
| Other Hispanic | 70（8.71） | 29（11.43） | 20（6.93） | 21（7.88） |  |
| Non-Hispanic White | 196（54.56） | 52（49.09） | 69（58.26） | 75（56.07） |  |
| Non-Hispanic Black | 122（9.47） | 27（6.00） | 39（8.54） | 56（14.11） |  |
| Non-Hispanic Asian | 110（6.27） | 44（7.34） | 40（6.32） | 26（5.11） |  |
| Other Race | 38（5.13） | 10（5.58） | 14（5.77） | 14（3.94） |  |
| Smoking，n（%） |  |  |  |  | 0.015 |
| Yes | 258（40.40） | 75（34.44） | 86（39.33） | 97（47.77） |  |
| No | 432（59.60） | 147（65.56） | 150（60.67） | 135（52.23） |  |
| Diabetes，n（%） |  |  |  |  | 0.018 |
| Yes | 151（17.93） | 44（16.68） | 48（13.89） | 59（23.78） |  |
| No | 539（82.07） | 178（83.32） | 188（86.11） | 173（76.22） |  |
| Significant fibrosis，n（%） |  |  |  |  | 0.187 |
| Yes | 107（15.65） | 38（16.44） | 36（12.42） | 33（18.47） |  |
| No | 583（84.35） | 184（83.56） | 200（87.58） | 199（81.53） |  |
| Alcohol intake（g） | 8.09±19.16 | 11.49±19.76 | 7.90±22.47 | 4.79±12.85 | 0.001 |
| BMI，（Kg/m³） | 33.75±6.43 | 32.58±5.66 | 33.50±6.27 | 35.24±7.05 | ＜0.001 |
| TC（mmol/L） | 5.05±0.97 | 4.92±0.91 | 5.12±1.00 | 5.13±1.00 | 0.009 |
| TG（mmol/L） | 1.99±2.10 | 1.97±1.76 | 2.04±1.89 | 1.94±2.69 | 0.904 |
| HDL-C（mmol/L） | 1.23±0.32 | 1.22±0.28 | 1.23±0.35 | 1.24±0.34 | 0.941 |
| HbA1c（%） | 5.92±1.22 | 5.82±1.00 | 6.04±1.41 | 5.89±1.24 | 0.168 |
| ALM_BMI_ | 0.76±0.18 | 0.84±0.17 | 0.74±0.17 | 0.70±0.17 | ＜0.001 |
| Sarcopenia，n（%） |  |  |  |  | 0.189 |
| No | 562（84.24） | 189（87.70） | 191（83.44） | 182（81.57） |  |
| Yes | 128（15.76） | 33（12.30） | 45（16.56） | 50（18.43） |  |

Comparison of DII by sarcopenia subgroups (weighted).

| Variable | Women | | | Men | | |
| --- | --- | --- | --- | --- | --- | --- |
|  | Non-sarcopenia | Sarcopenia | P-value | Non-sarcopenia | Sarcopenia | P-value |
| DII | 1.43±1.55 | 2.18±1.49 | 0.003 | 0.44±1.80 | 0.50±1.86 | 0.836 |

The association between DII and sarcopenia (weighted).

| Variable | Model 1 | Model 2 | Model 3 |
| --- | --- | --- | --- |
|  | OR（95%CI） P-value | OR（95%CI） P-value | OR（95%CI） P-value |
| Women |  |  |  |
| Continuous DII | 1.41（1.09，1.83） 0.010 | 1.57（1.20，2.05） 0.001 | 1.64（1.22，2.20） 0.001 |
| Categorical DII |  |  |  |
| T1 group | 1.00（reference） | 1.00（reference） | 1.00（reference） |
| T2 group | 1.47（0.54，4.03） 0.453 | 1.89（0.64，5.58） 0.251 | 1.54（0.46，5.16） 0.484 |
| T3 group | 2.34（0.89，6.14） 0.086 | 3.93（1.46，10.59） 0.007 | 3.87（1.31，11.42）0.014 |
| Men |  |  |  |
| Continuous DII | 1.02（0.82，1.25） 0.882 | 1.03（0.80，1.33） 0.819 | 1.01（0.78，1.32） 0.913 |
| Categorical DII |  |  |  |
| T1 group | 1.00（reference） | 1.00（reference） | 1.00（reference） |
| T2 group | 1.74（0.72，4.19） 0.219 | 2.19（0.86，5.56） 0.100 | 2.69（0.94，7.67） 0.065 |
| T3 group | 1.60（0.65，3.97） 0.310 | 1.47（0.45，4.83） 0.524 | 1.51（0.43，5.30） 0.520 |

Multivariate linear regression model between DII and ALMBMI (weighted)

| ALM_BMI_ | Model 1 | Model 2 |
| --- | --- | --- |
|  | β（95%CI） P-value | β（95%CI） P-value |
| Women | -0.009（-0.017，-0.002） 0.016 | -0.007（-0.014，-0.001） 0.039 |
| Men | -0.003（-0.010，0.005） 0.455 | -0.002（-0.009，0.006） 0.670 |
